# RSV versus SARS-CoV-2 bei Kindern: Vergleich stationärer Verläufe

**DOI:** 10.1007/s10354-025-01094-8

**Published:** 2025-09-01

**Authors:** Alexandra Victoria Brey, Florian Pengg, Thomas Frischer, Angela Zacharasiewicz

**Affiliations:** 1 Faculty for Medicine, Sigmund Freud Private University, Freudplatz 3, 1020 Vienna, Austria; 2 Department of Pediatrics and Adolescent Medicine, Klinik Ottakring, Vienna, Austria 1160

**Keywords:** RSV bei Kindern, SARS-CoV‑2 bei Kindern, Akute Atemwegsinfektionen bei Kindern, Vergleich SARS-CoV-2 Varianten, Vergleich RSV und SARS-CoV‑2, RSV in children, SARS-CoV‑2 in children, Acute respiratory infections in children, Comparison of SARS-CoV‑2 variants, Comparison of RSV and SARS-CoV‑2

## Abstract

SARS-CoV‑2 und RSV sind häufige virale Erreger akuter Atemwegserkrankungen. Während RSV bei Kindern unter zwei Jahren oft zu schweren Verläufen führt, sind diese bei Covid-19 selten. Die Studie analysierte 264 hospitalisierte Kinder (0–12 Jahre) mit SARS-CoV-2- oder RSV-Infektionen zwischen September 2021 und März 2022. RSV-Infektionen verliefen schwerer als SARS-CoV-2-Infektionen: 55 % der RSV-Patient*innen benötigten eine Sauerstofftherapie, im Vergleich zu 9,5 % der SARS-CoV-2-Fälle (*p* < 0,001). Die Sauerstofftherapiedauer lag bei RSV bei 2 ± 2,6 Tagen, bei SARS-CoV‑2 bei 0,3 ± 1,07 Tagen. RSV-Patient*innen benötigten signifikant länger eine O_2_-Therapie (*p* < 0,001) und einen längeren Krankenhausaufenthalt (*p* < 0,001) als SARS-CoV-2-Omikron-Betroffene. Im Vergleich zu Delta war nur die O_2_-Therapiedauer bei RSV signifikant länger (*p* < 0,001). Delta-Patient*innen hatten eine längere Hospitalisierungsdauer als Omikron-Patientinnen (*p* < 0,001).

## Abstract (en)

SARS-CoV‑2 and RSV are common viral pathogens causing acute respiratory infections. While RSV often leads to severe cases in children under two years, severe Covid-19 cases in children are rare. This study analysed 264 hospitalised children (0–12 years) with SARS-CoV‑2 or RSV infections between September 2021 and March 2022. RSV infections were more severe than SARS-CoV‑2 infections: 55% of RSV patients required oxygen therapy compared to 9.5% of SARS-CoV-2 cases (*p* < 0.001). The duration of oxygen therapy was 2 ± 2.6 days for RSV and 0.3 ± 1.07 days for SARS-CoV‑2. RSV patients required significantly longer O_2_ therapy (*p* < 0.001) and had longer hospital stays (*p* < 0.001) than SARS-CoV‑2 Omicron cases. Compared to Delta, only the O_2_ therapy duration was significantly longer in RSV cases (*p* < 0.001). Delta patients had longer hospital stays than Omicron patients (*p* < 0.001).

## Hintergrund

SARS-CoV‑2 und das Respiratorische Synzytialvirus (RSV) zählen zu den häufigsten Atemwegserregern und rufen ähnliche Symptome hervor [1]. Dennoch zeigt ein Vergleich der Publikationsanzahlen in medizinischen Datenbanken wie PubMed oder Cochrane, dass SARS-CoV‑2 auch mehr als vier Jahre nach Beginn der Pandemie und trotz Aufhebung des globalen Notstands im Mai 2023 [2] weiterhin deutlich mehr Beachtung erhält. So wurden allein im Jahr 2024 über zehnmal mehr Studien zu SARS-CoV‑2 veröffentlicht als zu RSV. RSV gilt jedoch als ernstzunehmender Erreger [3], der weltweit bei Säuglingen und Kleinkindern zu schweren Erkrankungen der unteren Atemwege führt [4]. In Deutschland ist es beispielsweise für mehr als die Hälfte aller Hospitalisierungen aufgrund schwerer Atemwegsinfektionen bei Kindern unter einem Jahr verantwortlich [5]. Obwohl es einige Untersuchungen gibt, in denen bereits Unterschiede zwischen Infektionen mit SARS-CoV‑2 und RSV bei hospitalisierten Kindern erforscht wurden [6–9], fehlen bisher Studien mit repräsentativen Fallzahlen, die den klinischen Verlauf von RSV- und SARS-CoV-2-Infektionen bei Kindern bis zwölf Jahren im selben Zeitraum und unter identischen stationären Bedingungen direkt vergleichen.

### RSV

Das Respiratorische Synzytialvirus ist der wichtigste Erreger wiederkehrender Atemwegsinfektionen bei Säuglingen und Kleinkindern [10]. Seine Relevanz ergibt sich sowohl aus den häufig resultierenden schweren Krankheitsverläufen [11] als auch aus seiner hohen Infektiosität [10].

Weltweit infizieren sich jährlich ungefähr 33 Mio. Kinder unter fünf Jahren mit RSV [12], von denen etwa jedes zehnte Kind eine stationäre Behandlung benötigt [4]. Dabei sind zirka vier von fünf der aufgrund von RSV hospitalisierten Kinder gesunde, reif geborene Säuglinge, die vorwiegend jünger als ein halbes Jahr sind [13–15]. Die Ansteckung geschieht dabei in der Regel über Tröpfcheninfektion oder durch Berührung von kontaminierten Gegenständen und Oberflächen [16], wobei RS-Viren etwa 20 min auf Händen und 45 min auf Papierhandtüchern und Baumwollstoffen infektiös bleiben können [17].

Aufgrund der sehr hohen Infektiosität können ab dem zweiten erlebten Winter bereits bei zirka 90 bis 100 % der Kinder RSV-Antikörper nachgewiesen werden. Jede erneute Infektion verläuft typischerweise weniger schwerwiegend als die erste, da sie bei Kindern, die älter als zwei Jahre alt sind, normalerweise nur den oberen Respirationstrakt betrifft [13]. Die Wahrscheinlichkeit für das Entwickeln schwerwiegender Symptome ist bei Neugeborenen und Säuglingen aufgrund der unvollständig entwickelten Atemwegsorgane und ihres schwächeren Immunsystems deutlich höher [18]. Weitere allgemeine Risikofaktoren für das Entwickeln eines schweren RSV-Verlaufs sind unter anderem Frühgeburtlichkeit, Kinder mit bronchopulmonalen Dysplasien, hämodynamisch signifikanten Herzfehlern, Immundefekten sowie chronischen pulmonalen Erkrankungen [17].

Zumeist entwickeln Kinder zu Beginn einer RSV-Infektion Krankheitszeichen, welche zunächst die oberen Atemwege betreffen. Im Verlauf kommt es oft zu weiteren, auch die unteren Atemwege betreffenden, Symptomen. Patient*innen, die im Rahmen ihrer RSV-Infektion eine Bronchiolitis entwickeln, präsentieren sich klinisch oft mit einem reduzierten Allgemeinzustand, verminderter Nahrungsaufnahme sowie Zeichen erhöhter Atemarbeit. Eine kausale Therapie [17] sowie einen zugelassenen RSV-Impfstoff zur aktiven Immunisierung gibt es im pädiatrischen Kontext derzeit nicht. Es existieren jedoch monoklonale Antikörper zur passiven Immunisierung, die für Neugeborene und Säuglinge ausdrücklich empfohlen sind [19].

### SARS-CoV-2

SARS-CoV‑2 (Schweres Akutes Atemwegssyndrom-Coronavirus Typ 2) ist das Virus, das die Coronavirus-Erkrankung 2019 (Covid-19) verursacht [20] und in weiterer Folge zur ersten Pandemie des 21. Jahrhunderts führte [21].

Zu den häufigsten Symptomen bei mit SARS-CoV‑2 infizierten Kindern zählen Fieber und produktiver Husten [22]. Gleichzeitig ist es möglich, dass die Betroffenen kaum oder gar keine Krankheitszeichen aufweisen. Die wichtigsten Risikofaktoren für einen schweren Verlauf einer SARS-CoV-2-Infektion umfassen Adipositas, Diabetes mellitus, Asthma bronchiale, angeborene Herzfehler sowie Erkrankungen, die das Nervensystem oder den Stoffwechsel beeinträchtigen [23]. Im Vergleich mit Erwachsenen verlaufen SARS-CoV-2-Infektionen bei Kindern normalerweise milder. Besonders gefährdet für einen schweren Verlauf und eine erhöhte Mortalität sind Kinder unter fünf Jahren, insbesondere Neugeborene [24]. Der häufigste Übertragungsweg ist die Aufnahme von virusbeladenen Partikeln über die Atemwege. Auch der Kontakt mit SARS-CoV-2-verunreinigten Oberflächen stellt ein Risiko für Ansteckung dar [25]. Ab dem vollendeten sechsten Lebensmonat besteht die Möglichkeit einer Schutzimpfung [19].

Die multisystemische Erkrankung Long COVID kann nach einer SARS-CoV-2-Infektion bei Kindern jeden Alters auftreten [26], wobei verschiedene Studien eine niedrigere Prävalenz während der Omikron-Periode im Vergleich zur Delta-Periode beschreiben [27, 28].

## Methoden

Die Daten dieser retrospektiven, monozentrischen Beobachtungsstudie wurden zwischen September 2021 und März 2022, unter Einhaltung der ethischen Standards der Deklaration von Helsinki, in der Abteilung für Kinder- und Jugendheilkunde eines großen städtischen Krankenhauses, der Klinik Ottakring in Wien, erhoben. Während dieses Zeitraums wurde bei sämtlichen ambulanten Patient*innen ein Nasen-Rachen-Abstrich zum Virusnachweis mittels Multiplex-PCR vorgenommen. In die Studie eingeschlossen wurden alle Kinder unter zwölf Jahren, die im Rahmen des PCR-Tests entweder positiv auf RSV oder SARS-CoV‑2 getestet wurden und aufgrund der Infektion stationär aufgenommen werden mussten. Die Indikationen zur Hospitalisierung waren Dehydratation, zunehmende Dyspnoe, Zeichen einer beginnenden respiratorischen Erschöpfung, Hypoxämie mit SpO_2_ < 92 % unter Raumluft, intermittierende Bradykardien, ausgeprägte Tachypnoe, persistierendes hohes Fieber trotz Antipyretika sowie schwere Begleiterkrankungen oder Risikofaktoren wie Frühgeburtlichkeit. Kinder mit einer gleichzeitigen Infektion beider Viren wurden von der Studie ausgeschlossen. Während des Krankenhausaufenthaltes wurden verschiedene klinische und laborchemische Parameter, wie die Leukozytenzahl, der CRP-Wert und die Dauer der Sauerstofftherapie, erfasst, um einen Vergleich zwischen RSV- und SARS-CoV-2-Patient*innen zu ermöglichen.

Zur Analyse signifikanter Unterschiede zwischen den Gruppen wurde die Software SPSS verwendet. Nominale Parameter wurden mithilfe von t‑Tests oder Mann-Whitney-U-Tests geprüft, ordinale Variablen mit Kreuztabellen und Chi-Quadrat-Tests. Das Signifikanzniveau wurde per Bonferroni-Korrektur von 0,05 auf 0,005 angepasst, um Fehler erster Art zu minimieren. Für die Subgruppenanalysen (Vergleich RSV, SARS-CoV‑2 Delta und SARS-CoV‑2 Omikron) ergab die Bonferroni-Korrektur einen adjustierten Signifikanzwert von 0,002.

Auf eine Fallzahlplanung wurde verzichtet, da die Stichprobengröße der Anzahl der während des Studienzeitraums stationär aufgenommen Patient*innen entsprach. Vor Beginn der Untersuchung wurde ein positives Votum der Ethikkommission der Stadt Wien eingeholt (EK 23-064-VK).

## Resultate

Insgesamt wurde der Multiplex-PCR-Test bei 1242 ambulanten Patient*innen durchgeführt. Davon wurden bei 666 Patient*innen entweder SARS-CoV-2- oder RSV-Infektionen diagnostiziert. Während 409 (61,4 %) der Kinder mit SARS-CoV‑2 infiziert waren, waren 257 (38,6 %) RSV-positiv. Bei zwölf (1,8 %) Kindern wurde gleichzeitig eine Infektion mit SARS-CoV‑2 und mit RSV diagnostiziert. 264 der 666 mit SARS-CoV‑2 oder RSV infizierten Kinder mussten aufgrund ihrer Erkrankung hospitalisiert werden (*n* = 264). Die deskriptiven Daten dieser 264 stationären Patient*innen sind in Tab. 1 aufgelistet. Bei 169 (64 %) der hospitalisierten Patient*innen wurde RSV nachgewiesen und 95 (36 %) der Kinder waren mit SARS-CoV‑2 infiziert. Während das Geschlechtsverhältnis bei den SARS-CoV-2-Patient*innen annährend ausgeglichen ist (49,5 % männlich, 50,5 % weiblich), zeigten sich bei den RSV-Infizierten deutlich mehr Buben (62,1 %) als Mädchen (37,9 %). Das durchschnittliche Alter lag bei den SARS-CoV-2-Patient*innen bei 14 Monaten und war damit minimal, jedoch nicht signifikant niedriger als bei den RSV-Infizierten (16 Monate) (*p* = 0,478).

**Tab. 1:** Charakteristiken der 264 stationären Patient*innen: Deskriptive Daten

Patient*innen	RSV (*n* = 169)	SARS-CoV‑2 (*n* = 95)	Σ (*n* = 264)	*p*-Wert
Männlich	105 (62,1 %)	47 (49,5 %)	152 (57,6 %)	–
Weiblich	64 (37,9 %)	48 (50,5 %)	112 (42,4 %)	
***Alter***				
Durchschnittliches Alter (in Monaten)	16	14	15	0,478
Neugeborene	16 (9,5 %)	13 (13,7 %)	29 (11 %)	
Säuglinge	93 (55,0 %)	51 (53,7 %)	144 (54,5 %)	
Kleinkinder	23 (13,6 %)	14 (14,7 %)	37 (14 %)	
Vorschulkinder	35 (20,7 %)	13 (13,7 %)	48 (18,2 %)	
Schulkinder	2 (1,2 %)	4 (4,2 %)	6 (2,3 %)	
***Frühgeburten***				
Anteil der Frühgeburten	13 (7,7 %)	3 (3,2 %)	16 (6,1 %)	0,138
***Zugrundeliegende Vorerkrankungen***				
Durchschnittliche Anzahl an Vorerkrankungen	0,2	0,2	0,2	0,939
Keine Vorerkrankung	141 (83,4 %)	81 (85,3 %)	222 (84,1 %)	
Eine Vorerkrankung	23 (13,6 %)	9 (9,5 %)	32 (12,1 %)	
Zwei Vorerkrankungen	5 (3 %)	5 (5,3 %)	10 (3,8 %)	

Definitionen: Neugeborene < 1 Monat | Säuglinge 1–12 Monate | Kleinkinder 13–24 Monate, Vorschulkinder 25–72 Monate | Frühgeburt: Geburt vor der 37. SSW

Die Medianwerte und p‑Werte der Mann-Whitney-U- und t‑Tests sind in Tab. 2 dargestellt. Hospitalisierte RSV- und SARS-CoV-2-Patient*innen unterschieden sich signifikant in der Dauer der O_2_-Therapie und des Krankenhausaufenthalts (jeweils *p* < 0,001). Bezüglich Alter, Frühgeburtlichkeit, Dauer der Körpertemperatur über 38 °C, Dauer der intravenösen Therapie sowie der CRP- und Leukozytenwerte bei Aufnahme zeigten sich keine signifikanten Unterschiede zwischen den beiden Gruppen.

**Tab. 2:** Mediane der klinischen Ergebnisse aller 264 Kinder der Studienpopulation nach Kohorte

Merkmal	RSV (*n* = 169)	SARS-CoV‑2 (*n* = 95)	*p*-Wert
Alter zum Zeitpunkt des PCR-Tests (in Monaten)	4,0 [1,0–23,5]	8,0 [2,0–17]	0,381
Körpertemperatur > 38 °C (in Tagen)	0,0 [0,0–2,0]	0,0 [0,0–1,0]	0,931
CRP bei Aufnahme (in mg/L)	6,8 [2,9–22,5]	3,4 [2,9–11,0]	0,081
*O*_2_-Therapie (in Tagen)	1,0 [0,0–3,0]	0,0* [0,0–0,0]	**<** **0,001**
Intravenöse Therapie (in Tagen)	0,0* [0,0–0,0]	0,0* [0,0–0,0]	0,720
Leukozyten bei Aufnahme (pro $\mathrm{\mu}l$)	9,73 [7,8–14,3]	8,7 [6,6–11,5]	0,010
Hospitalisierung (in Tagen)	4,0 [3,0–6,0]	3 [2,0–4,0]	**<** **0,001**
Frühgeburten (vor der 37. SSW)	13	3	0,138

Werte in eckigen Klammern entsprechen jeweils den Werten der 25- und 75-Quartile; *Median inklusive Quartile = 0 aufgrund durchschnittlich sehr kurzer Therapiedauern

### Dauer der O_2_-Therapie und Hospitalisierung

**Abb. 1:**
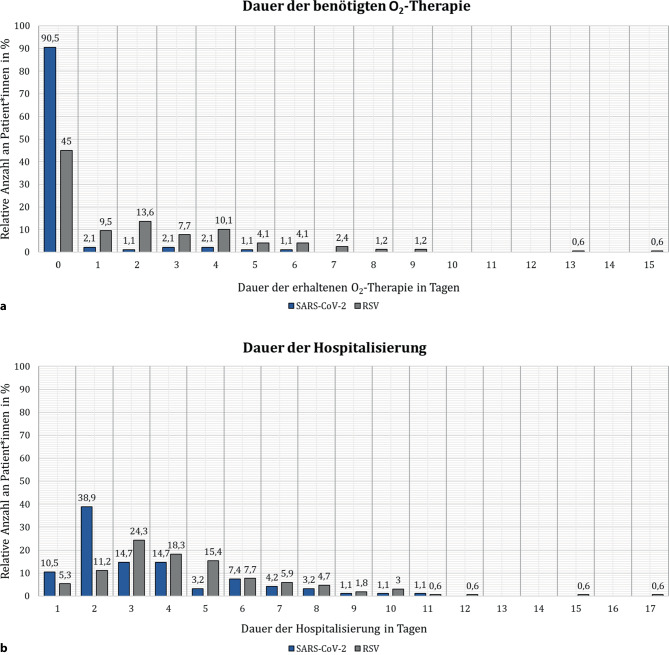
Dauer der benötigten Sauerstofftherapie (**a**) und Hospitalisierung (**b**) bei SARS-CoV-2- und RSV-Patient*innen

Deutlich mehr RSV-Kinder benötigten zusätzlichen Sauerstoff (*p* < 0,001) als SARS-CoV-2-Betroffene. Sie erhielten diesen durchschnittlich auch über einen längeren Zeitraum als SARS-CoV-2-Patient*innen (Abb. 1a). Während 55 % aller 169 RSV-Infizierten zusätzlichen Sauerstoff benötigten, bedurften nur 9,5 % aller 95 SARS-CoV-2-Kinder eine Sauerstofftherapie. Die durchschnittliche Dauer dieser betrug bei RSV-Patient*innen zwei Tage (± 2,6 d). Kinder, die mit SARS-CoV‑2 infiziert waren, benötigten durchschnittlich lediglich 0,3 Tage zusätzlichen Sauerstoff (± 1,07 d). Weiters unterschied sich die benötigte Hospitalisierungsdauer zwischen den Gruppen signifikant (*p* < 0,001) (Abb. 1b). Die durchschnittliche Aufenthaltsdauer der mit RSV diagnostizierten Kinder betrug 4,5 Tage (± 2,56 d). Patient*innen, die mit SARS-CoV‑2 infiziert waren, blieben typischerweise nur 3,4 Tage in der Klinik (± 2,16 d).

### Vergleich RSV, SARS-CoV-2 Delta und SARS-CoV-2 Omikron

Da im Datensatz keine Unterscheidung zwischen den SARS-CoV-2-Varianten Delta (B.1.617.2) und Omikron (B.1.1.529) vorgenommen wurde, erfolgte eine post hoc Differenzierung. Kinder, die vor dem 27.12.2021, jenem Zeitpunkt, an dem sich Omikron als dominante SARS-CoV-2-Variante in Österreich etablierte und Delta als dominante Variante ablöste [29], mit SARS-CoV‑2 diagnostiziert wurden, wurden als Delta-Patient*innen klassifiziert, während Kinder, die nach diesem Datum positiv getestet wurden, als Omikron-Proband*innen gelten. Nach dieser Methode besuchten im Studienzeitraum insgesamt 65 Delta- und 344 Omikron-infizierte Kinder die Ambulanz. Von diesen benötigten 31 Delta- und 64 Omikron-Patient*innen eine stationäre Behandlung.

**Abb. 2:**
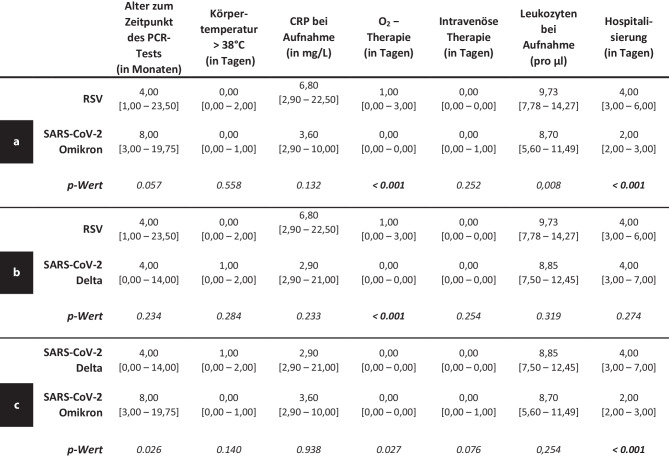
Vergleich der Medianwerte der klinischen Parameter, inklusive Quartile, zwischen RSV und SARS-CoV‑2 Omikron (3a), RSV und SARS-CoV‑2 Delta (3b) sowie SARS-CoV‑2 Delta und SARS-CoV‑2 Omikron (3c)

In den Mann-Whiney-U-Tests zeigen sich signifikante Differenzen zwischen RSV und SARS-CoV‑2 Omikron bei der Dauer der Sauerstofftherapie (*p* < 0,001) sowie der Dauer der Hospitalisierung (*p* < 0,001). Die RSV-Patient*innen benötigten im Durchschnitt sowohl eine längere O_2_-Therapie als auch einen längeren stationären Aufenthalt (Abb. 2a). Im Vergleich zwischen RSV und SARS-CoV‑2 Delta zeigt sich lediglich ein signifikanter Unterschied bezüglich der Dauer der O_2_-Therapie (*p* < 0,001), wobei die RSV-Patient*innen länger zusätzlich Sauerstoff benötigten (Abb. 2b). Der Vergleich zwischen den beiden SARS-CoV-2-Varianten zeigt, dass lediglich die Dauer der Hospitalisierung (*p* < 0,001) signifikant unterschiedlich war. Die Delta-Infizierten benötigten dabei im Durchschnitt einen längeren stationären Aufenthalt (Abb. 2c).

## Diskussion

Die Ergebnisse dieser Studie stehen im Einklang mit den Resultaten früherer Untersuchungen.

Ähnlich wie in den Publikationen von Fedorczak et al. [6], Meyer et al. [7] und Ozdemir et al. [8] zeigt auch die vorliegende Studie einen signifikant längeren Spitalsaufenthalt von RSV-Patient*innen als bei SARS-CoV-2-Infizierten. Zwar variieren die exakten Aufenthaltsdauern zwischen den Studien, doch es zeigt sich ein einheitliches Bild einer signifikant längeren Hospitalisation bei RSV als bei SARS-CoV‑2 infizierten Kindern. Dies weist auf tendenziell schwerwiegendere Verläufe und die Notwendigkeit einer intensiveren medizinischen Betreuung bei RSV-Infizierten hin. Auch hinsichtlich des Bedarfs einer Sauerstofftherapie zeigen sich deutliche Übereinstimmungen mit früheren Studien. In einer retrospektiven Analyse von Balas et al. wurde festgestellt, dass Kinder mit RSV häufiger Dyspnoe, schwere Hustenanfälle, pathologische Auskultationsbefunde und Nasenobstruktionen aufweisen als Covid-19-Patient*innen [9]. Sowohl bei Fedorczak et al. (*p* < 0,001) [6] als auch bei Ozdemir et al. (*p* = 0,03) [8] benötigten RSV-Patient*innen signifikant häufiger eine Sauerstoffgabe als SARS-CoV-2-Infizierte – ein Befund, der in der vorliegenden Studie erneut bestätigt wurde (*p* < 0,001). Diese Ergebnisse deuten darauf hin, dass RSV-Infektionen bei Kindern häufiger zu schweren Atemwegssymptomen führen, die eine Sauerstofftherapie erforderlich machen. Der erhöhte Sauerstoffbedarf könnte zudem auf ein erhöhtes Risiko für schwerwiegendere Komplikationen wie Bronchiolitis oder Pneumonie hinweisen. Infolgedessen führen sowohl der signifikant längere O_2_-Therapiebedarf als auch die prolongierte Krankenhausaufenthaltsdauer bei RSV-Infizierten zu einer höheren Belastung des Gesundheitssystems im Vergleich zu SARS-CoV-2-Infektionen im Kindesalter.

Die Zahl der mit SARS-CoV‑2 diagnostizierten Kinder war während der Delta-Welle (65 Fälle) mehr als fünfmal geringer als nach dem Dominieren von Omikron (344 Fälle). Von diesen insgesamt 409 Kindern war bei 31 Delta- und 64-Omikron-Infizierten eine Hospitalisierung indiziert. Da die beiden Zeiträume annähernd gleich lang waren suggerieren die Ergebnisse, wie auch in anderen Untersuchungen von unter anderem Setiabudi et al. [30] und Wang et al. [31] beschrieben, dass Omikron die ansteckendere Variante zu sein scheint und daher auch zu mehr Krankenhausaufenthalten führt. Ähnlich wie in den Ergebnissen von Wang et al. [31] deuten auch die Daten dieser Studie darauf hin, dass Omikron-Infektionen, beispielsweise aufgrund der kürzeren Hospitalisierungsdauer, mit etwas milderen Verläufen einhergehen als Delta-Infektionen. Die signifikant längere O_2_-Therapie und der prolongierte Krankenhausaufenthalt bei RSV-Infizierten im Vergleich zu SARS-CoV‑2 Omikron deuten darauf hin, dass RSV schwerwiegendere Atemwegsbeschwerden verursacht. Der fehlende signifikante Unterschied in der Hospitalisierungsdauer zwischen RSV und SARS-CoV‑2 Delta könnte darauf hindeuten, dass die Delta-Variante ebenfalls mit schwereren Verläufen assoziiert ist als Omikron. Zudem bestätigt der Vergleich zwischen Delta und Omikron, dass die Delta-Variante zu längeren Krankenhausaufenthalten führte, was mit früheren Studien übereinstimmt [31].

### Stärken und Limitationen

Die vorliegende Arbeit weist diverse Stärken auf, wie das Setting in dem die Daten erhoben wurden. Da alle Patient*innen im selben Krankenhaus und ebenfalls an derselben Abteilung behandelt wurden, kann eine konsistente Versorgung angenommen werden und mögliche Störfaktoren, die auf potenzielle Unterschiede in der Behandlung zurückzuführen sind, werden minimiert. Sämtliche Patient*innen hatten Zugang zu demselben Team an Ärzt*innen und Pflegepersonen, die nach den gleichen Behandlungsrichtlinien arbeiten. Dadurch wurden mögliche Einflüsse unterschiedlicher Behandlungsmethoden eliminiert, was eine hohe Vergleichbarkeit sowie eine hohe Validität der Ergebnisse gewährleistet. Zweitens zeigen sich weder beim Alter der Patient*innen noch bei den zugrundeliegenden Vorerkrankungen oder der Anzahl an Frühgeburten signifikante Differenzen zwischen den SARS-CoV-2- und den RSV-Infizierten. Dadurch wird die Aussagekraft der Ergebnisse gestärkt, da die beobachteten Unterschiede eher auf die Infektion selbst als auf individuelle Patient*inneneigenschaften zurückzuführen sind. Zudem hat die Klinik dieser Untersuchung ein großes Einzugsgebiet, das Patient*innen aus diversen Kulturen und Ländern umfasst. Das bedeutet, dass die Patient*innenpopulation dieser Studie vielfältig ist und die daraus resultierenden Ergebnisse extrapolierbar sind.

Diese Studie umfasst nur hospitalisierte Patient*innen, sodass mildere Fälle von pädiatrischen RSV- und SARS-CoV-2-Infektionen, die keine Hospitalisierung erforderten, nicht berücksichtigt wurden. Den behandelnden Ärzt*innen und Krankenpfleger*innen waren die Diagnosen der Kinder bei Hospitalisierung bekannt. Ein Information-Bias kann daher nicht gänzlich ausgeschlossen werden. Für den Vergleich der Schweregrade von RSV und SARS-CoV‑2 wurden jedoch nicht nur durch die fehlende Randomisierung potenziell beeinflusste Parameter, wie beispielweise die Aufenthaltsdauer in Tagen, sondern verschiedenste klinische, nicht beeinflussbare, Aspekte miteinander verglichen. Zu den hiervon nicht betroffenen Parametern zählen unter anderem die Leukozytenwerte im Labor und die Dauer des Fiebers in Tagen. Performance Bias kann aufgrund der sehr ähnlichen Rahmenbedingungen bezüglich Handling und Therapie der beiden Erkrankungen, wie gleiche Isolationsmaßnahmen und symptomatische Behandlung aufgrund des Fehlens einer wirksamen kausalen Therapie [17, 24, 32], weitestgehend ausgeschlossen werden. Da in der herangezogenen Datenbank kein Eintrag vorliegt, wie viele Kinder zusätzlich zur Sauerstofftherapie eine Atemunterstützung, beispielsweise mittels High-Flow-Sauerstofftherapie, erhielten, ist die Aussagekraft zum Therapieaufwand eingeschränkt. Weiters gibt es im Datensatz bei mit SARS-CoV‑2 infizierten Kindern keine Angaben, ob diese mit den SARS-CoV-2-Varianten Delta oder Omikron infiziert waren. Um diesen Nachteil zu relativieren wurde post-hoc zwischen den Varianten unterschieden. Diese Vorgehensweise ermöglichte eine grobe Unterscheidung zwischen den beiden SARS-CoV-2-Varianten und half, die Schweregrade der Varianten in gewissem Maße zu berücksichtigen. Obwohl diese post-hoc Differenzierung zwar eine Verbesserung darstellt, weist sie dennoch Einschränkungen auf, da mit hoher Wahrscheinlichkeit manche Kinder nicht der richtigen SARS-CoV-2-Variante zugeordnet werden konnten.

## Schlussfolgerung

Stationäre RSV-Patient*innen unter zwölf Jahren benötigten signifikant häufiger und länger eine Sauerstofftherapie sowie einen längeren Krankenhausaufenthalt als SARS-CoV-2-Infizierte (jeweils *p* < 0,001). Im Vergleich mit SARS-CoV-2-Omikron wiesen RSV-Patient*innen ebenfalls längere O_2_-Therapie- und Hospitalisierungsdauern auf (je *p* < 0,001). RSV-Infizierte benötigten im Vergleich zu SARS-CoV-2-Delta-Patient*innen eine längere O_2_-Therapie (*p* < 0,001). Der Vergleich zwischen Delta und Omikron ergab signifikant längere Krankenhausaufenthalte für Delta-Infizierte (*p* < 0,001).

## Abkürzungen

Abs.: Absolut
CRP: C‑reaktives Protein
d: Days (Deutsch: Tage)
m: Months (Deutsch: Monate)
O_2_: Sauerstoff
PCR: Polymerasekettenreaktion
Rel.: Relativ
RSV: Respiratorisches Synzytial Virus
SARS-CoV‑2: Severe acute respiratory syndrome coronavirus 2 (Deutsch: Schweres akutes respiratorisches Syndrom Coronavirus 2)
SD: Standardabweichung
SSW: Schwangerschaftswoche
